# Induced in vitro adaptation for salt tolerance in date palm (*Phoenix dactylifera* L.) cultivar Khalas

**DOI:** 10.1186/s40659-020-00305-3

**Published:** 2020-08-26

**Authors:** Suliman A. Al-Khateeb, Abdullatif A. Al-Khateeb, Muhammad N. Sattar, Akbar S. Mohmand

**Affiliations:** 1 Department of Environment Natural Resources, College of Agriculture and Food Sciences, King Faisal University, P.O. Box 400, Al-Ahsa, 31982 Kingdom of Saudi Arabia; 2 Department of Agriculture Biotechnology, College of Agriculture and Food Sciences, King Faisal University, P.O. Box 400, Al-Ahsa, 31982 Kingdom of Saudi Arabia; 3 Central Laboratories, King Faisal University, Box 420, Al-Ahsa, 31982 Saudi Arabia; 4 Research, Innovation and Commercialization (ORIC), Bacha Khan University Charsadda, Charsadda, Khyber Pakhtunkhawa Pakistan

**Keywords:** Date palm, Tissue culture, Salt stress, Ion relation, K^+^/Na^+^, Photosynthesis, Regenerants

## Abstract

**Background:**

Soil salinity causes huge economic losses to agriculture productivity in arid and semiarid areas worldwide. The affected plants face disturbances in osmotic adjustment, nutrient transport, ionic toxicity and reduced photosynthesis. Conventional breeding approaches produce little success in combating various stresses in plants. However, non-conventional approaches, such as in vitro tissue culturing, produce genetic variability in the development of salt-tolerant plants, particularly in woody trees.

**Results:**

Embryogenic callus cultures of the date palm cultivar Khalas were subjected to various salt levels ranging from 0 to 300 mM in eight subcultures. The regenerants obtained from the salt-treated cultures were regenerated and evaluated using the same concentration of NaCl with which the calli were treated. All the salt-adapted (SA) regenerants showed improved growth characteristics, physiological performance, ion concentrations and K^+^/Na^+^ ratios than the salt non-adapted (SNA) regenerants and the control. Regression between the leaf Na^+^ concentration and net photosynthesis revealed an inverse nonlinear correlation in the SNA regenerants. Leaf K^+^ contents and stomatal conductance showed a strong linear relationship in SA regenerants compared with the inverse linear correlation, and a very poor coefficient of determination in SNA regenerants. The genetic fidelity of the selected SA regenerants was also tested using 36 random amplified polymorphic DNA (RAPD) primers, of which 26 produced scorable bands. The primers generated 1–10 bands, with an average of 5.4 bands per RAPD primer; there was no variation between SA regenerants and the negative control.

**Conclusion:**

This is the first report of the variants generated from salt-stressed cultures and their potential adaptation to salinity in date palm cv. Khalas. The massive production of salt stress-adapted date palm plants may be much easier using the salt adaptation approach. Such plants can perform better during exposure to salt stress compared to the non-treated date palm plants.

## Background

Worldwide, agriculture is affected by a number of biotic and abiotic stresses, including salinity [13, 45]. The inhibitory effects of soil salinity include osmotic stress, nutritional disparities and ion toxicity, resulting in the reduction of photosynthesis and other physiological disorders [58]. Moreover, salt stress is considered a principal abiotic stress and has been the focus of in vitro selection and applications [51]. Currently, these methods represent a key complement to classical breeding methods [61].

It has consistently been a challenging task to develop plants with improved economic yield and performance under various abiotic stresses [20]. Variations in growth and yield response have been reported in a number of species such as fruit crops [19] and potato [36] to develop salt-tolerant lines. However, the complex nature of salinity stresses makes investigations more challenging. Inter-species and inter-genera transfer of salt stress tolerance traits through conventional breeding have been employed with little success to improve the genetic base of crop plants against salt stress [51].

Among the nonconventional breeding methods, tissue and cell culture offer great promises for crop improvement against salt stress [22, 25]. Many researchers have described drought and salt-tolerant lines and regenerated plants using in vitro techniques in a number of plant species [1, 12, 16, 22]. However, the genotypic differences of the plants regenerated from the calli also depends on the source of the explant used [26].

Date palm (*Phoenix dactylifera* L.) tree belongs to the Palmaceae (Arecaceae) family and inhabits tropical and subtropical habitats, particularly the Middle East. Date palm is tolerant to a number of abiotic stresses such as salinity, sodicity, drought and high temperature [59]. However, variation in tolerance is observed among different varieties/cultivars growing within an area [4, 40]. The tissue culture technique has been widely used for mass commercial production of date palm through date palm off-shoot micropropagation. Additionally, several physiological studies related to environmental stresses [7] and plant responses [7, 14] have also been conducted in date palm. Al-Khateeb and Al-Khateeb [7] found significant differences among five date palm cultivars in salinity tolerance by studying Na^+^, Cl^−^, Ca^2+^ and K^+^ accumulation. While studying salinity in date palm, Al-Khateeb and Al-Khateeb [9] also observed the K^+^/Na^+^ ratio in the growth medium plays an important role in ameliorating the adverse effects of salinity. These results reflect the possibility of using an in vitro technique for further evaluating the date palm cultivars under salinity stress. The ability of cultured cells to adapt to salinity is widespread among plant species and is expressed at the tissue and whole plant level [36]. Date palm plants have the potential to gradually adapt to salinity, which would be lethal if the exposure is sudden [11]. The introduction of abiotic and biotic stress tolerance traits through somaclonal variation is economically very important in date palm [29]. It may also help to widen the genetic base of date palm to cope with salinity stress. The roles of different biochemical, physiological and DNA-based molecular markers are very significant in the estimation of somaclonal variations during in vitro culturing [2, 48]. DNA-based molecular markers, including restriction fragment length polymorphisms (RFLPs and amplified fragment length polymorphisms (AFLPs, are routinely used, however, the requirement for expensive restriction enzymes and radiolabeled probes make these techniques unsuitable for routine diagnostics [17]. Comparatively, random amplified polymorphic DNA (RAPD) markers provide a rapid, cheaper and reliable technique to identify genetic changes during in vitro culturing [56].

Keeping in mind the potential of the methodology to obtain potential stress-tolerant variants, the aims of the present studies were to (i) obtain regenerated salt-tolerant plants by subjecting the calli of date palm cv. Khalas to various salt levels and (ii) evaluate these regenerants for different morphological, physiological parameters and genetic stability.

## Materials and methods

These studies were undertaken at the Department of Agriculture Biotechnology and the Department of Environment and Natural Resources, College of Agriculture and Food Sciences, King Faisal University, Saudi Arabia.

### Callus induction

Embryogenic calli (1000 mg for each treatment) were obtained from the date palm cultivar Khalas using the method previously described by Al-Khateeb and Al-Khateeb [9]. The callus cultures were transferred to the basal media for plant regeneration as described by Al-Khateeb and Al-Khateeb [8] and root induction as described by Al-Khateeb [5].

### In vitro salt selection

The embryogenic calli were grown in 0, 100, 200, 300, and 400 mM sodium chloride (NaCl) in Murashige and Skoog medium (MS-medium) [46]. Calli were subjected to two protocols to induce stress. One portion of the calli (four replicates in each treatment) was transferred directly to 0, 100, 200, 300 and 400 mM NaCl. The other portion was subjected to a stepwise treatment with various concentrations of NaCl (four replicates in each treatment). The increment started with 50 mM salt treatment every passage for 4 weeks. The salt level was increased by 50 mM, i.e., from 0 to 50, 50 to 100, 100 to 150, 150 to 200, 200 to 250 and 250 to 300 mM every passage over a four-week duration. The data for 400 mM NaCl treatment was not included due to completely impaired growth of the embryogenic calli. After six passages, the embryogenic calli were cultured on regeneration medium without NaCl to obtain the regenerants. No calli survived due to direct exposure to high concentration of NaCl, and therefore, only the results for the stepwise increments are presented and discussed.

### Plant rooting and regeneration

The salt-selected calli were then subjected to regeneration medium as explained by Al-Khateeb and Al-Khateeb [8] to obtain plantlets from the respective salt treatments. The obtained regenerants were then subjected to salt stress at the respective salt levels of 100, 200 and 300 mM for eight weeks to test their salt tolerance at the whole plant level. Two types of regenerants were obtained: regenerants obtained from calli treated with salt in stepwise increments, named salt-adapted (SA) regenerants, regenerants obtained from NaCl-free medium and treated with the same salt concentrations, named salt non-adapted (SNA) regenerants**.** In total, twelve regenerants were obtained for each group of SA and SNA regenerants, respectively.

### Gas exchange analysis

Photosynthesis (A), transpiration rate (E), stomatal conductance (g_**s**_) and intercellular CO_2_ (C_i_) were measured from two leaves of four different plants per treatment using an Infra-Red Gas analyser (CI-301 CO_2_ Gas Analyzer, CID Inc. USA) and calculated according to von Caemmerer and Farquhar [57]. Mesophyll conductance (g_m_), which is a composite measure of all the liquid phase conductance of CO_2_ (cell wall, plasmalemma, cytoplasm, chloroplast membrane) as well as of the conductance associated with carboxylation [18] was calculated as follows: g_m_ = A/C_i_ [24]. The water use efficiency (WUE) was calculated using the following formula: WUE = A/E.

### Ion relation analysis

Plant parts were separated into shoots and roots. The shoots were washed twice in distilled water. The ions were removed from the free spaces around the roots as described by Al-Khateeb et al. [9] for two min in isotonic sorbitol solution for each of the various treatment concentrations. Fresh leaves and roots of approximately 500 mg were homogenized using a mortar and pestle and extracted in a 25-ml volumetric flask in distilled deionized water at 90 °C for four hrs. The Na^+^ and K^+^ in leaves of the SA and SNA regenerants were determined using a GBS 905 Atomic Absorption Spectrophotometer (Shamatzo Inc., Japan) and expressed as relative values. For Na^+^, the final diluted solutions contained 0.1 KCl to control ionization, while for Ca^+^ and Mg^+^, the final diluted solutions contained 1% lanthanum chloride to reduce interference. The nutrient composition results were calculated and presented on a fresh water value basis. To determine the dry weights, leaves and roots were dried at 85 °C for 48 h.

### Total genomic DNA isolation

Total genomic DNA was isolated from micropropagated SA plantlets under three NaCl treatments, i.e., 100 mM, 200 mM and 300 mM, including control plants, using a plant DNA-miniprep protocol-modified from Arif et al. [15]. Briefly, 100–150 mg of freshly harvested date palm leaves were placed in a sterile mortar. Sterile sand (100 mg) and 500 µl of lysis buffer (0.1 M Tris–HCl (pH 8.0), 0.05 M EDTA, 0.5 M NaCl and 0.01 M β-mercaptoethanol) were added, and the plant material was finely crushed using a mortar and pestle. The crushed leaf material was transferred to a sterile Eppendorf tube (1.5 ml), followed by an additional 1000 µl of lysis buffer and vigorous vortexing. The leaf extracts were then incubated at ~ 65 °C for 30 min with occasional mixing. After incubation at room temperature, the tubes were centrifuged at 12,000 rpm for 5 min. The supernatant (~ 200 µl) was carefully transferred to a new tube, and an equal volume of chloroform:isoamyl alcohol (24:1) was added. The tubes were mixed well with gentle shaking and centrifuged at 12,000 rpm for 5 min at room temperature. The supernatant (200 µl) was transferred to a new tube, and the DNA was precipitated with cold isopropanol (500 µl). All tubes were kept at − 80 °C for 30 min followed by centrifugation at 12,000 rpm for 10 min. All the supernatant was discarded, and the DNA pellet was washed with 70% cold ethanol. The pellet was air-dried at room temperature, and the DNA was dissolved in 50 µl MilliQ water. The DNA was quantified, and the quality was analyzed by separating 2.0 µl of total genomic DNA by 1% agarose gel electrophoresis in 0.5 TAE buffer. The DNA was stored at − 20 °C for further downstream applications.

### RAPD-PCR analysis of genomic DNA

RAPD amplification was performed in a total reaction volume of 25 µl including template DNA (~ 50–100 ng), 2.5 µl of 10 pmol of 10-mer oligo-deoxynucleotide primer (Operon Technologies, Alameda, California) (Table-6), 2.5 µl of dNTPs (0.4 mM each), DreamTaq DNA polymerase (ThermoScientific) and 2.5 µl DreamTaq buffer (10x). PCR was performed in a thermal cycler (Bio-Rad) with initial denaturation at 95 °C for 5 min, 35 cycles of 95 °C for 35 s, 36 °C for 35 s, 72 °C for 2 min, and a final extension at 72 °C for 5 min. The PCR products were separated in a 1% agarose gel using 0.5× TAE buffer. The sizes of all amplicons were confirmed by comparison to a 1 kb DNA ladder (Takara).

### Statistical analysis

A completely randomized design was used with seven treatments and four replications per treatment. Analysis of variance was used to observe the significance between mean values. Duncan's multiple range test (DMRT) was used to compare the means at P < 0.05 [55]. SAS Statistical software (SAS, 2011) was used for all statistical analyses. The relationships between the selected characters were analyzed using simple nonlinear regression analysis available in Microsoft Excel.

## Results

### Selection of regenerants

In this experiment, four replicates for each treatment were used for morphological, physiological and biochemical studies. Four plants each were obtained as control and all other treatments, respectively from embryogenic calli grown in salt-free MS medium.

### Response of regenerants to NaCl stress (morphological characters)

Significant variations were observed in most of the morphological and physiological characters (Table 1). The data revealed that SNA regenerants produced more roots than SA regenerants in response to three NaCl levels, i.e., 100, 200 and 300 mM. The maximum number of roots were obtained only for SNA regenerants at 100 mM NaCl compared to the control. The SA regenerants produced maximum number of leaves (4.5) at 200 and 300 mM NaCl compared to the SNA regenerants (Table 1). Similarly, the SA regenerants produced significantly longer leaf lengths at 300 mM NaCl compared to the SNA regenerants and the control. The SNA regenerants had a greater leaf thickness than SA regenerants; however, with an increase in the salinity level to 300 mM, the leaf thickness of the SA regenerants was improved compared with the SNA regenerants.

**Table 1:** Morphological characters of regenerants of the date palm cv. Khalas subjected to salt stress after regeneration from salt-stressed cultures

Regenerants/characters	Salt levels (mM NaCl)	Number of roots	Number of leaves	Leaf length (cm	Leaf thickness
Control	0	4.0 ± 1.8b	3.5 ± 0.6bc	13.3 ± 1.3c	0.75 ± 0.24a
Salt Non-adapted (SNA)	100	5.3 ± 0.5a	3.0 ± 0.0 cd	24.4 ± 2.4b	0.58 ± 0.06b
	200	3.8 ± 0.9b	4.0 ± 0.8ab	25.6 ± 4.5b	0.62 ± 0.11ab
	300	2.8 ± 0.5bc	2.8 ± 0.5d	25.2 ± 4.5b	0.34 ± 0.6c
Salt adapted (SA)	100:100	1.5 ± 0.6d	3.0 ± 0.0 cd	28.5 ± 3.0ab	0.29 ± 0.03c
	200:200	2.8 ± 0.5bc	4.5 ± 0.6a	26.5 ± 3.6ab	0.30 ± 0.04c
	300:300	1.8 ± 0.5 cd	4.5 ± 0.6a	30.8 ± 5.2a	0.36 ± 0.06c

Means followed by the same letter(s) are not significantly different at the 5% level of probability

### Response of regenerants to NaCl stress (growth characteristics)

The growth characteristics of both the SNA and SA regenerants improved significantly in response to 200 and 300 mM NaCl as compared to 100 mM NaCl. However, the SA regenerants performed better than the SNA regenerants, with very consistent results at all salinity levels. The root dry weight of SA regenerants consistently showed greater improvement compared with the SNA regenerants. The SA regenerants showed improved growth, as evidenced based on most of the growth parameters used at the respective salt concentrations (Table 2).

**Table 2:** Growth characteristics of regenerants of the date palm cv. Khalas subjected to salt stress after regeneration from salt-stressed cultures

Regenerants/characters		Shoot dry weight (g)	Root dry weight (g)	Total dry weight (g)	Dry weight shoot/root ratio
Control	0	0.795 ± 0.181a	0.365 ± 0.117a	1.160 ± 0.253a	2.18 ± 0.661a
Salt non-adapted (SNA)	100	0.222 ± 0.022c	0.180 ± 0.018d	0.403 ± 0.040e	1.23 ± 0.002b
	200	0.690 ± 0.075a	0.289 ± 0.051bc	0.980 ± 0.094b	2.44 ± 0.423a
	300	0.460 ± 0.082b	0.306 ± 0.055ab	0.766 ± 0.137c	1.50 ± 0.001b
Salt adapted (SA)	100:100	0.267 ± 0.029c	0.241 ± 0.026bcd	0.509 ± 0.054de	1.11 ± .0.001b
	200:200	0.300 ± 0.042c	0.231 ± 0.032 cd	0.531 ± 0.073de	1.30 ± 0.001b
	300:300	0.328 ± 0.055c	0.292 ± 0.049bc	0.620 ± 0.104 cd	1.12 ± 0.001b

Means followed by the same letter(s) are not significantly different at the 5% level of probability

### Response of regenerants to NaCl stress (biochemical characters)

#### Leaf ion concentrations

The effect of salt stress was also determined for the leaf ions Na^+^, K^+^, Ca^+^, and Mg^+^ and the K^+^/Na^+^ ratio in both SNA and SA regenerants (Table 3). Significant variations were observed in the SNA regenerants and SA regenerants for Na^+^ accumulation under salt stress conditions. All SA regenerants accumulated less Na^+^ under salt stress conditions compared with the respective SNA regenerants at all NaCl concentrations (Table 3). A gradual decline was also observed in K^+^, Ca^+^ and Mg^+^ concentrations in all SA regenerants compared with SNA regenerants. The minimum K^+^ (33.1 µgg^−1^) was recorded in SA regenerants compared to 88.6 µgg^−1^ K^+^ in SNA regenerants at 300 mM NaCl (Table 3). A similar trend was also observed for the Ca^+^ and Mg^+^ contents.

**Table 3:** Leaf mineral contents of regenerants of the date palm cv. Khalas subjected to salt stress after regeneration from the same salt-stressed cultures

Regenerants/characters	NaCl (mM)	Na^+^	K^+^	Ca^2+^	Mg^2+^	K^+^/Na^+^ ratio
Control	0	70.8 ± 11.90f	102.3 ± 12.1a	75.8 ± 7.9b	30.0 ± 3.6a	1.482 ± 0.324a
Salt non-adapted (SNA)	100	198.6 ± 8.90d	83.9 ± 3.4b	141.6 ± 5.8a	7.1 ± 0.3bc	0.422 ± 0.000bc
	200	324.0 ± 16.4b	64.4 ± 3.3c	63.9 ± 3.2c	5.4 ± 0.3c	0.199 ± 0.000d
	300	536.3 ± 18.1a	88.6 ± 3.0b	139.8 ± 4.7a	7.4 ± 0.2b	0.165 ± 0.000d
Salt adapted (SA)	100:100	173.8 ± 4.90e	42.3 ± 2.8e	36.5 ± 2.4d	8.0 ± 0.5b	0.573 ± 0.001b
	200:200	236.5 ± 14.3c	54.2 ± 3.3d	62.2 ± 3.8c	6.3 ± 0.4bc	0.229 ± 0.000 cd
	300:300	240.8 ± 9.00c	33.1 ± 1.2f	33.4 ± 1.2d	6.2 ± 0.2bc	0.137 ± 0.000d

Means followed by the same letter(s) are not significantly different at the 5% level of probability

The highest K^+^/Na^+^ ratio (1.482) was observed in the control plants compared with all the SNA regenerants and SA, while the lowest 0.14 K^+^/Na^+^ ratio was recorded in the SA regenerants at 300 mM NaCl (Table 3). The K^+^/Na^+^ ratios of SA regenerants were significantly higher compared with the SNA regenerants in the presence of 100 and 200 mM NaCl.

#### Root ion concentrations

The root Na^+^, K^+^, Ca^2+^ and Mg^2+^ contents were also determined in SNA and SA regenerants at all salinity levels. An increasing trend was observed for the accumulation of Na^+^ contents in SNA and SA regenerants with increasing salinity. All SA regenerants accumulated lower Na^+^ contents in their roots compared to SNA regenerants (Table 4).

**Table 4:** Root mineral contents of regenerants of the date palm cultivar Khalas subjected to salt stress after regeneration from salt-stressed cultures

Regenerants/characters	NaCl (mM)	Na^+^	K^+^	Ca^+^	Mg	K^+^/Na^+^ ratio
Control	0	62.5 ± 7.5f	88.5 ± 6.4a	88.0 ± 7.3b	14.0 ± 2.6a	1.432 ± 0.204a
Salt non-adapted (SNA)	100	90.2 ± 3.7d	44.3 ± 1.8e	84.5 ± 4.7c	5.6 ± 0.2f	0.491 ± 0.000c
	200	113.4 ± 5.7c	85.3 ± 4.4ab	73.5 ± 3.7d	10.1 ± 0.5b	0.752 ± 0.000b
	300	160.7 ± 5.4a	54.4 ± 1.8d	90.5 ± 3.0a	6.0 ± 0.2e	0.339 ± 0.000d
Salt adapted (SA)	100:100	82.1 ± 5.5e	68.0 ± 4.5c	50.1 ± 3.3e	4.4 ± 0.3 g	0.828 ± 0.001b
	200:200	112.2 ± 6.8c	81.9 ± 4.9b	49.1 ± 3.0e	9.1 ± 0.6c	0.730 ± 0.000b
	300:300	125.8 ± 4.7b	67.9 ± 2.6c	28.8 ± 1.1f	7.7 ± 0.3d	0.539 ± 0.000c

Means followed by the same letter(s) are not significantly different at the 5% level of probability

In contrast, the K^+^, Ca^2+^ and Mg^2+^ concentrations were significantly reduced with increasing salinity levels in both SNA and SA regenerants. The K^+^ and Mg^+^ concentrations were significantly higher in the roots of SA compared with SNA regenerants at all salinity levels. However, the Ca^+^ accumulation was significantly enhanced in the roots of SNA than SA regenerants under the same salinity levels (Table 4).

The K^+^/Na^+^ ratio was significantly greater in SA compared with SNA regenerants at all salinity levels. The K^+^/Na^+^ ratio in the roots of SNA regenerants was dramatically reduced with increasing salinity. Comparatively, the SA regenerants maintained a consistent K^+^/Na^+^ ratio under increasing salt concentrations, showing better performance than the SNA regenerants.

### Response of regenerants to NaCl stress (gas exchange capacity)

The effect of salinity on the photosynthesis (A), transpiration rate (€), stomatal conductance (g_s_), mesophyll conductance (g_m_) and internal CO_2_ concentration (C_i_) was also studied. The data revealed that increasing salt levels significantly decreased the photosynthesis, transpiration rate, water use efficiency (WUE) and stomatal conductance (Table 5) in SNA regenerants and SA regenerants.

**Table 5:** In vitro performance assessment of adapted and non-adapted regenerants for various physiological parameters

Regenerants/characters	NaCl (mM)	Photosynthesis rate (µmol/m^2^/s)	Transpiration rate (mmol/m^2^/s)	Stomatal conductance (mmol/m^2^/s) (g_s_)	Mesophyll conductance (mmol/m^2^/s) (g_m_)	Internal CO_2_ (µmol/mol	WUE (µmol/mmol
Control	0	6.13 ± 0.80a	1.35 ± 0.233a	37.58 ± 14.13a	44.7 ± 11.4a	142.0 ± 29.9bc	4.60 ± 0.74d
Salt non-adapted (SNA)	100	4.79 ± 0.56bc	0.74 ± 0.111b	26.98 ± 4.32b	33.3 ± 9.2ab	148.1 ± 23.7bc	6.49 ± 0.22 cd
	200	3.87 ± 1.46c	0.47 ± 0.210 cd	21.75 ± 6.28bc	19.8 ± 9.6bc	205.7 ± 30.8b	9.87 ± 5.83bc
	300	4.24 ± 0.17c	0.34 ± 0.058de	11.15 ± 2.44d	11.6 ± 3.9c	389.9 ± 95.2a	13.03 ± 2.90b
Salt adapted (SA)	100:100	4.48 ± 0.51c	0.69 ± 0.060bc	15.45 ± 2.33 cd	40.0 ± 3.3a	111.9 ± 9.1c	6.53 ± 0.73 cd
	200:200	5.65 ± 0.44ab	0.58 ± 0.190bcd	17.90 ± 7.93bcd	41.0 ± 17.4a	160.8 ± 69.0bc	10.37 ± 2.92bc
	300:300	4.64 ± 0.46bc	0.21 ± 0.029e	13.35 ± 1.05 cd	32.1 ± 2.4ab	210.7 ± 13.1b	22.55 ± 4.41a

Means followed by the same letter(s) are not significantly different at the 5% level of probability

The SA regenerants showed a significantly better photosynthetic rate, stomatal conductance and mesophyll conductance than the SNA regenerants (Table 5). The stomatal and mesophyll conductance increased significantly with increasing salinity in the SA regenerants and vice versa compared to the SNA regenerants. The transpiration rate and internal CO_2_ were significantly higher in the SNA regenerants compared with the SA regenerants. At the highest salinity level (300 mM NaCl), the intercellular CO_2_ concentration of SNA and SA regenerants was 389.9 and 210.7 µmol/mol, respectively. Similarly, the stomatal and mesophyll conductance responded differently in SNA and SA regenerants. The stomatal conductance of SNA regenerants was 11.15 mmol/m^2^/s at 300 mM NaCl compared to 13.35 mmol/m^2^/s for SA regenerants. Similarly, the mesophyll conductance of SNA and SA regenerants was 11.6 and 32.1 mmol/m^2^/s at 300 mM NaCl, respectively (Table 5). Generally, the SA regenerants maintained consistent mesophyll and stomatal conductance even under high salt concentrations compared to SA regenerants.

The WUE of SA and SNA regenerants increased significantly with increasing salinity as compared to control plants, but SA regenerants performed much better than SNA regenerants. The WUE of SA regenerants increased from 6.53 µmol/mol at 100 mM NaCl to 22.55 µmol/mol at 300 mM NaCl compared to SNA regenerants (Table 5).

### Correlation analysis

**Fig. 1:**
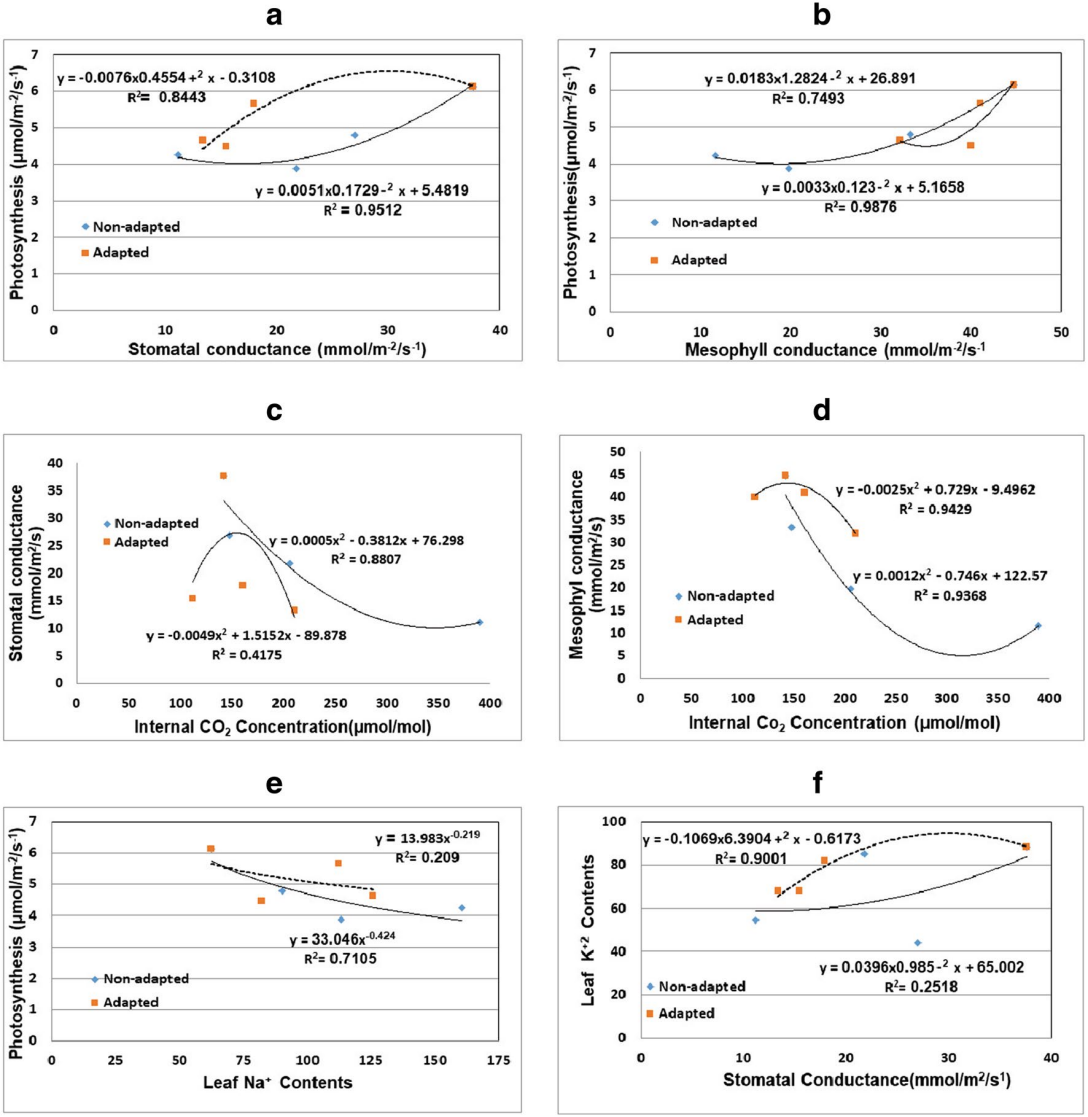
Regression analysis between **(A)** photosynthesis and stomatal conductance, **(B)** photosynthesis and mesophyll conductance, **(C)** internal CO_2_ concentration and stomatal conductance, **(D)** internal CO_2_ concentration and mesophyll conductance, **(E)** mean Na^+^ contents and photosynthesis rate and **(F)** mean K^+^ contents and stomatal conductance in adapted and non-adapted regenerants of the date palm cv. Khalas

Correlations were computed between photosynthesis, stomatal and mesophyll conductance, internal CO_2_ (Ci) and leaf Na^+^ and K^+^ contents for the SNA and SA regenerants of cv. Khalas (Fig. 1). The correlation coefficients for the photosynthetic rate with stomatal and mesophyll conductance were determined for SNA regenerants (r^2^ = 0.8443, r^2^ = 0.7493) and SA regenerants (r^2^ = 0.9512, r^2^ = 0.9876) (Fig. 1), respectively. A regression between the leaf dry matter Na^+^ concentration and net photosynthesis rate provided an inverse nonlinear correlation with a good coefficient of determination r^2^ of 0.71 in non-adapted regenerants and a poor r^2^ of 0.21 in adapted regenerants (Fig. 1e). The correlation coefficients between K^+^ contents and stomatal conductance were r^2^ = 0.9001 in SA regenerants and r2 = 0.2518 in SNA regenerants.

### RAPD-PCR analysis

**Fig. 2:**
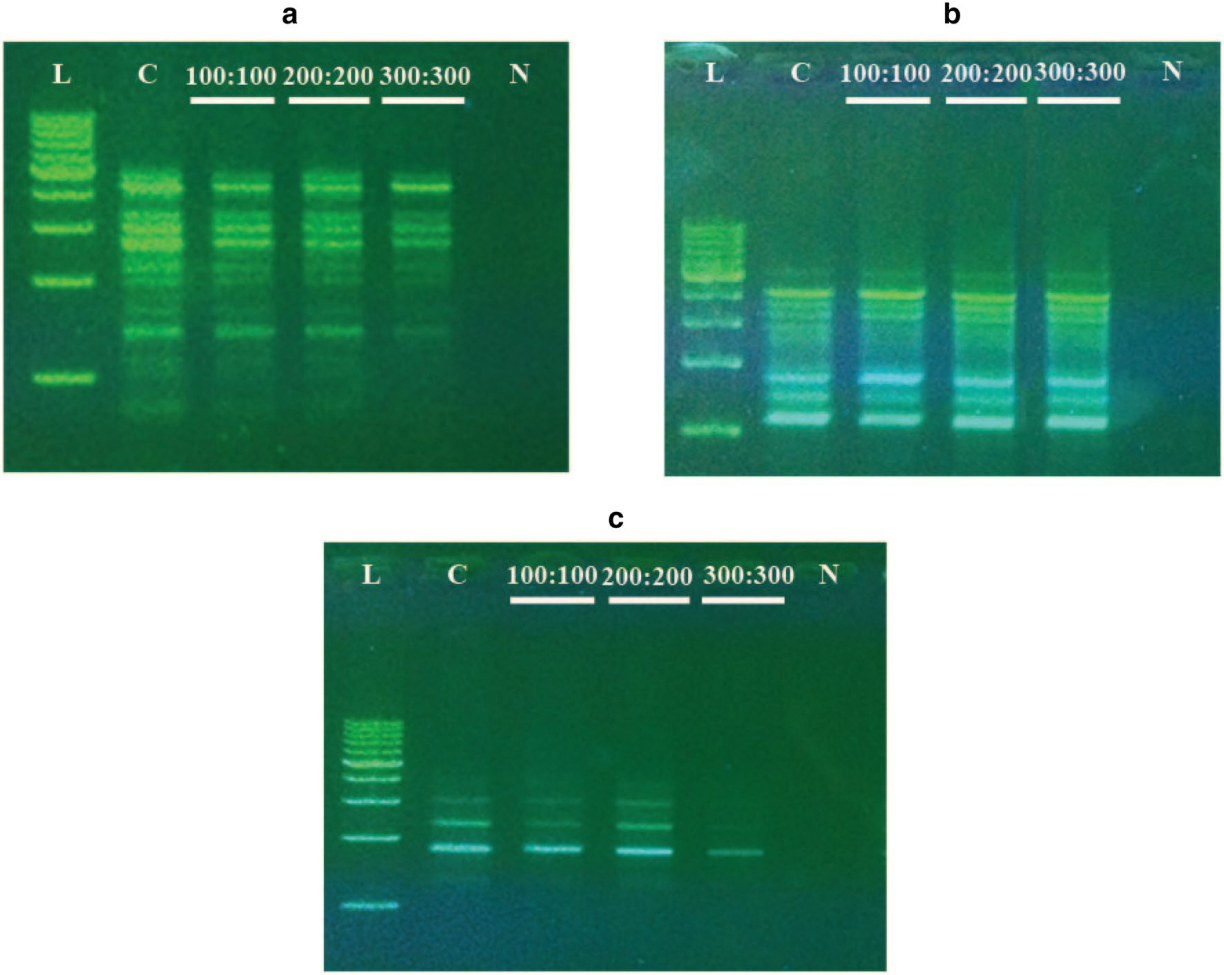
PCR amplification of regenerated salt-adapted date palm plants of the cultivar ‘Khalas’ under three salinity levels, i.e., 100, 200 and 300 mM NaCl, using primers **a** Moh-05, **b** OPA-10 and **c** OPC-05. From left–right, lane 1–6 represents 1-kb DNA ladder (Takara), control plant, 100, 200, and 300 mM NaCl and negative PCR control

To test the genetic fidelity of the selected regenerants against salt stress, 36 primers were tested, and 26 primers produced scorable bands (Table 6). These 26 primers produced 140 amplicons for each treatment, ranging between 250–6000 bp in size. The selected primers generated 1–10 bands with an average of 5.4 bands per RAPD primer (Fig. 2). In total, 560 bands (total number of plants analyzed x total number of amplicons with all primers) were generated using RAPD, revealing a monomorphic pattern in all regenerated plants.

**Table 6:** List of RAPD primers used to detect genetic variation in the date palm cultivar Khalas under in vitro salt stress conditions

No	Primer name	Primer sequence	Band score				Size range (bp)
			Control	100 mM	200 mM	300 mM	
1	OPA-03	AGTCAGCCAC	9	9	9	9	500–4000
2	OPA-05	AGGGGTCTTG	6	6	6	6	500–2700
3	OPA-07	GAAACGGGTG	6	6	6	6	750–7000
4	OPA-10	GTGATCGCAG	7	7	7	7	1200–5000
5	OPA-15	TTCCGAACCC	1	1	1	1	4000
6	OPB-10	CTGCTGGGAC	10	10	10	10	800–6000
7	OPC-02	GTGAGGCGTC	1	1	1	1	2000
8	OPC-05	GATGACCGCC	3	3	3	3	1800–3100
9	OPC-08	TGGACCGGTG	9	9	9	9	700–4000
10	OPE-01	CCCAAGGTCC	6	6	6	6	250–2700
11	OPE-18	GGACTGCAGA	4	4	4	4	1200–3000
12	OPE-19	ACGGCGTATG	8	8	8	8	600–2800
13	OPF-10	GGAAGCTTGG	2	2	2	2	900–2000
14	OPH-04	GGAAGTCGCC	2	2	2	2	3000–3900
15	OPI-02	GGAGGAGAGG	4	4	4	4	800–3600
16	OPI-08	TTTGCCCGGT	5	5	5	5	750–4800
17	OPM-10	TCTGGCGCAC	7	7	7	7	700–4000
18	OPN-13	AGCGTCACTC	2	2	2	2	1800–4000
19	OPR-13	GGACGACAAG	4	4	4	4	750–3000
20	OPW-11	CTGATGCGTG	3	3	3	3	750–2200
21	MOH-2	GAGGCGTCGC	5	5	5	5	1600–4500
22	MOH-3	CCCTACCGAC	3	3	3	3	1800–4000
23	MOH-5	CACCTTTCCC	5	5	5	5	1100–4000
24	MOH-7	GTTCCGCTCC	9	9	9	9	750–5000
25	MOH-8	GTGAGGCGTC	10	10	10	10	800–5000
26	MOH-9	GGACCCAACC	9	9	9	9	800–3600

## Discussion

The tissue culture technique is routinely employed to produce genetically stable and useful genetic variations in various plant species through somaclones. Plants thus regenerated undergo somaclonal variations, most likely as a result of stresses enforced during in vitro culturing [32].

Significant variations were observed in most of the morphological, physiological and biochemical characters in almost all the SA regenerants compared with the SNA regenerants. Physiological characters, such as shoot and root dry weight and shoot/root dry weight ratio, were first reduced and then tended to improve with high salinity in SA compared with SNA regenerants (Table 2). Such reductions have been previously reported by many researchers [3, 6]. The higher dry weights of SNA compared with SA regenerants might be a result of higher ion accumulation than organic compound accumulation, particularly in the presence of 200 and 300 mM NaCl. Such reductions could be associated with a reduction in some morphological characters of these regenerants (Table 1). Adverse effects of increasing salinity levels were more pronounced in leaves than roots; however, compared with the low salinity levels, the SA regenerants seemed to start responding to the high salinity with improved performance (Tables 1, 2).

It is generally believed that a greater accumulation of K^+^ leads to better tolerance to elevated Na^+^ in plants [37]. Nevertheless, the efficiency of plants to cope with increased salt stress is primarily dependent on the cytosolic K^+^/Na^+^ ratio rather than shoot and root Na^+^ and/or K^+^ accumulation capabilities [13]. For example, Egea et al. [23] and Genc et al. [28] have shown that higher accumulation of Na^+^ in salt-tolerant tomato or Na^+^ exclusion in salt-tolerant bread wheat have no relationship to their salt tolerance, respectively. Thus, the salt tolerance of plants is a direct measure of the intracellular K^+^/Na^+^ ratio in plants. Apparently, plants attempt to adjust their cytosolic K^+^/Na^+^ ratio via either reduced accumulation of Na^+^ or overcoming the loss of K^+^ [27]. Keeping these features in mind, the leaf and root ionic concentrations were also determined based on the Na^+^, K^+^, Ca^+^, Mg^+^ and K^+^/Na^+^ ratios of all the treatments to assess the role of K^+^ in salt stress tolerance of the date palm cultivar “Khalas”.

During this study, the SA regenerants showed a reduced accumulation of Na^+^ in leaves and roots compared with the SNA regenerants and the control. Moreover, K^+^ and Ca^2+^ levels were lower in SA than SNA regenerants; however, all the SA regenerants maintained a constant level of these elements both in leaves and roots. Increasing leaf and root internal Na^+^ concentrations have also been reported in tomato [31], sugarcane [25, 49], wheat [28, 58] and potato [33], as we observed in SNA regenerants. A declining trend was observed in K^+^, Ca^2+^ and Mg^2+^ levels in the leaves of SA compared with SNA regenerants. Usually, NaCl causes two types of effects, i.e., lowering the water potential (osmotic stress) and increasing Na^+^ (ionic toxicity). The presence of high salt concentrations in the growing medium or soil inhibits water absorption and minerals such as K^+^ and Ca^2+^ [13]. In contrast, Na^+^ and Cl^−^ exert their deleterious effects directly by entering the plant cells [51]. The SNA regenerants appeared to accumulate more K^+^ and Ca^+^ than the SA regenerants to stabilize osmosis to maintain metabolic functions.

The K^+^/Na^+^ ratio is an established criteria for assessing the salt tolerance of plants [28, 45, 59]. Date palm cultivars maintain a higher K^+^/Na^+^ ratio to salt stress with a positive correlation between the yield and K^+^/Na^+^ ratio [9]. In the present study, the SA regenerants accumulated less Na^+^ than the SNA regenerants and maintained a reasonable K^+^/Na^+^ ratio under salt stress conditions (Tables 3 and 4). Thus, we presumed that the SA regenerants adapted to the salt stress at the cellular level, which was expressed at the whole plant level. Many researchers have also supported that the K^+^/Na^+^ ratio depicts the salt tolerance of plants [21, 54]. Moreover, Shabala and Cuin [54] confirmed that the ability of plant cells to cope with higher salt stress depends on the cytosolic K^+^/Na^+^ ratio rather than the accumulation of Na^+^ and/or K^+^ contents in shoots and roots.

The photosynthetic rate and stomatal and mesophyll conductance was significantly increasing in SA compared with SNA regenerants under increased salinity levels (Table 5). However, the transpiration rate and internal CO_2_ was significantly higher in SNA than SA regenerants. This increased accumulation of intercellular CO_2_ might be a result of a low level of photosynthesis and low transpiration rate under high salt concentrations. Lower rates of photosynthesis at higher salinity levels were more pronounced in SNA than SA regenerants and were directly associated with a decrease in both stomatal and mesophyll conductance. However, in SA regenerants, the net photosynthesis appeared to be associated with a decrease in stomatal conductance, while the biochemical properties of the photosynthetic apparatus in the mesophyll cells did not seem to exhibit a negative response. In the presence of a lower stomatal conductance and intercellular CO_2_ concentration, stomatal closure might be the main reason for decreased photosynthetic rate in both SNA and SA regenerants. However, once the SA regenerants were acclimatized to the saline environment, their net photosynthesis and stomatal conductance improved significantly, i.e., at 300 mM NaCl (Table 5). The higher photosynthetic rate in the SA regenerants could also be explained by a reduced accumulation of internal CO_2_ in SA compared with SNA regenerants. Despite the differences in stomatal conductance between SNA and SA regenerants, a relatively constant rate of transpiration was observed. The transpiration rate might contribute to adequate delivery of ions into leaves, which might be used in osmotic adjustment and hence have no effect on plant growth [41]. The superior WUE of SA regenerants might be due to the adaptation of SA regenerants to high salt levels by maintenance of turgor and hence better growth than SNA regenerants (Table 5). Tolerance to salinity is usually associated with maintenance of the net photosynthetic rate and stomatal conductance [41]. We also found that the SA regenerants maintained a fair photosynthetic rate, which was associated with consistent stomatal and mesophyll conductance even under high salinity levels, compared with their respective SNA regenerants. However, the reduction in photosynthetic activity might also be associated with low stomatal conductance under salt stress [42, 60], or to a direct effect of Na^+^ as observed in woody citrus plants [38].

Photosynthesis was positively correlated with stomatal and mesophyll conductance both in SNA and SA regenerants. Lucia et al. [39] also observed decreased stomatal conductance with decreased photosynthesis in the conifer *Dacrydium cupressinum*. These findings corroborate with those of Al-Khateeb [10] and Yaish et al. [60], who observed considerable changes in g_m_ consistent with the changes in net photosynthesis expressed on either a leaf area or chlorophyll basis. Similar observations have also been reported by Redondo-Gomez et al. in *Atriplex portulacoides* [50] under salt stress. However, according to Qiu et al. [47], there is no evidence of the inhibition of photosynthesis in response to salinity.

The correlation between net photosynthesis and leaf Na^+^ contents in SNA regenerants indicates that Na^+^ affects net photosynthesis rates, while in SA regenerants, the Na^+^ may be regulated at an earlier time to negatively affect the photosynthetic rate. Leaf K^+^ contents and stomatal conductance in SA regenerants showed a strong nonlinear relationship compared with the inverse nonlinear correlation in SNA regenerants. This adaptive character seems to be the outcome of a better K^+^/Na^+^ ratio in SA regenerants rather than Na^+^ exclusion and/or K^+^ accumulation (Table 4). These findings are also consistent with the recent observations of Shabala and Cuin [54] advocating that the K^+^/Na^+^ ratio is a key determinant of plant salt tolerance [21, 54].

Based on physiological, morphological and biochemical analyses, a genetic change was expected as somaclonal variation in the SA regenerants. Therefore, molecular characterization of SA regenerants was also investigated using RAPD DNA markers. However, due to the short exposure to salt stress, SNA regenerants were not employed in the molecular analysis. The RAPD analysis produced only a monomorphic pattern in all SA regenerants, and no polymorphic bands were observed. In total, 560 bands were generated from 26 primers in this study, with no polymorphisms. These findings are comparable to the results of previous studies using various plant species and employing PCR-based RAPD analysis [30, 35], including date palm [34, 43]. The different physiological responses of SA regenerants were more likely related to RNA rather than DNA responses. It is possible that these differences were related to the magnitude of the response and not to inherited changes in SA regenerants. The regeneration and selection of salt stress adapted date palm plants would be potentially useful in the field for the massive production of salt-adapted plants. Thus, we may presume from our studies that plants that are generated using this technique would be more resistant to saline conditions than non-treated date palm plants. In addition to major changes at the chromosomal level or the duplication and deletion of specific regions, abiotic stress tolerance in SA regenerants may also arise due to single base changes in the respective genes. Thus, the variation in adaptation against salt stress is a physiological rather than a genetic phenomenon, which has also been observed in date palm cv. Sukary [11]. Moreover, the changes that occurred in regenerants may either be due to the effects of certain hormones and growth regulators in the culture medium, or they may be consequences of an alleviation of salt stress and of time during the long culturing period in SA regenerants. To detect any changes at the gene level, such as point mutations, further whole-genome sequencing studies and/or application of new breeding tools (NBTs) [53] may provide better insight into the in vitro stress response of SA regenerants in the future and help to devise better strategies against salt stress in date palm. Further studies on these regenerants at both the molecular level (by sequencing the gene portions involved) and the whole plant level may be helpful.

## Conclusion

Although the SA regenerants showed a positive response to the high salinity levels, this response may not have been related to any genetic changes incurred during salt stress because no polymorphisms were observed by RAPD analysis. Thus, the improved performance of SA regenerants may involve physiological phenomena related to salt-adaptation. Further, long-term field trials may also be necessary for assessing the real quality of in vitro produced woody plants. However, the long life cycle of woody plants may have drawbacks in the selection and assessment of the variability generated in regenerants, even during development or in subsequent generations. Thus, any changes (genetic or physiological) observed in the regenerants of woody plants might be difficult to assess without employing modern OMICS and genetic approaches.

## Data Availability

Not applicable.

## Abbreviations

AFLP: amplified fragment length polymorphism
DMRT: duncan’s multiple range test
C_i_: intercellular Co_2_
MS-media: murashige and Skoog media
A: photosynthesis
RAPD: random amplified polymorphic DNA
RFLP: restriction fragment length polymorphism
SNA: salt non-adapted
SA: salt-adapted
Gs: stomatal conductance
E: transpiration rate
WUE: water use efficiency

## References

[1] Abul-Soad AA, Al-Khayri JM, Jain S, Gupta P (2018). Date palm somatic embryogenesis from inflorescence explant. Step wise protocols for somatic embryogenesis of important woody plants. Forestry sciences.

[2] Ahmad R, Anjum MA, Malik W (2018). Characterization and evaluation of mango germplasm through morphological, biochemical, and molecular markers focusing on fruit production: an overview. Mol Biotechnol.

[3] Al-Abdoulhadi IA, Dinar HA, Ebert G, Büttner C (2011). Effect of salinity on leaf growth, leaf injury and biomass production in date palm (*Phoenix dactylifera* L.) cultivars. Indian J. Sci. Technol..

[4] Al Kharusi L, Assaha D, Al-Yahyai R, Yaish M (2017). Screening of date palm (*Phoenix dactylifera* L.) cultivars for salinity tolerance. Forests.

[5] Al-Khateeb AA (2001). Influence of different carbon sources and concentations on the root formation of date palm (*Phoenix dactylifera* L.) cv. Khanezi Zagazig J Agric Res.

[6] Alkhateeb A (2008). Comparison effects of sucrose and date palm syrup on somatic embryogenesis of date palm (*Phoenix dactylifera* L.). Am J Biotechnol Biochem.

[7] Al-Khateeb A, Al-Khateeb S (2007). Study and comparision of tolerance of different date palm (*Phoenix dactylifera* L.) cultivars to salinity under callus conditions. Eco Summit.

[8] Al-Khateeb A, Al-Khateeb S (2016). In Vitro role of hormones at multiplication stage of date palm (*Phoenix dactylifera* L.) cvs Khalas and Sukary. Res J Biotechnol.

[9] Al-Khateeb A, Al-Khateeb S (2015). Effect of different combinations of growth hormones and its interaction on callogenesis. Res J Biotechnol.

[10] Al-Khateeb S (1997). Effects of NaCl and Na2SO4 on growth, ion relations, water relations, and gas exchange of two *Atriplex* species.

[11] Al-Khateeb SA, Al-Khateeb AA, Sattar MN, Mohmand AA, El-Beltagi HS (2019). Assessment of somaclonal variation in salt-adapted and non-adapted regenerated date palm (*Phoenix dactylifera* L.). Fresen Environ Bull.

[12] Al-Khayri JM, Jain S, Gupta P (2018). Somatic embryogenesis of date palm (*Phoenix dactylifera* L.) from shoot tip explants. Step wise protocols for somatic embryogenesis of important woody plants. Forestry sciences.

[13] Almeida DM, Oliveira MM, Saibo NJM (2017). Regulation of Na^+^ and K^+^ homeostasis in plants: towards improved salt stress tolerance in crop plants. Genetic Mol Biol.

[14] Alvarez I, Tomaro ML, Benavides MP (2003). Changes in polyamines, proline and ethylene in sunflower calluses treated with NaCl. Plant Cell Tissue Organ Cult.

[15] Arif IA, Bakir MA, Khan HA, Ahamed A, Al Farhan AH, Al Homaidan AA, Al Sadoon M, Bahkali AH, Shobrak M (2010). A simple method for DNA extraction from mature date palm Leaves: Impact of sand grinding and composition of lysis buffer. Int J Mol Sci.

[16] Benderradji L, Bouzerzour H, Ykhlef N, Djekoun A, Kellou K (2007). Réponse à la culture in vitro de trois variétés de l’olivier (*Olea europaea* L.). Sci Technol.

[17] Bertaccini A, Paltrinieri S, Contaldo N, Musetti R, Pagliari L (2019). Standard detection protocol: PCR and RFLP analyses based on 16S rRNA gene. Phytoplasmas. Methods in molecular biology.

[18] Bradford KJ, Hsiao TC (1982). Physiological responses to moderate water stress. Physiological plant ecology II.

[19] Caboni E, Anselmi S, Donato E, Manes F (2003). In vitro selection of actinidia deliciosa clones tolerant to nacl and their molecular and in vivo ecophysiological characterisation. Acta Hortic.

[20] Chen Z, Newman I, Zhou M, Mendham N, Zhang G, Shabala S (2005). Screening plants for salt tolerance by measuring K+ flux: a case study for barley. Plant Cell Environ.

[21] Colmer TD, Flowers TJ, Munns R (2006). Use of wild relatives to improve salt tolerance in wheat. J Exp Bot.

[22] Dennis T, Sreejesh K (2004). Callus induction and plant regeneration from cotyledonary explants of ash gourd (*Benincasa hispida* L.). Sci Horticult..

[23] Egea I, Pineda B, Ortíz-Atienza A (2018). The SlCBL10 calcineurin B-like protein ensures plant growth under salt stress by regulating Na^+^ and Ca^2+^ homeostasis. Plant Physiol.

[24] Fites JA, Teskey RO (1988). CO 2 and water vapor exchange of Pinus taeda in relation to stomatal behavior: test of an optimization hypothesis. Can J For Res.

[25] Gandonou C, Abrini J, Idaomar M, Skali Senhaji N (2005). Response of sugarcane (*Saccharum* sp.) varieties to embryogenic callus induction and in vitro salt stress. Afr J Biotechnol.

[26] Ganeshan S, Båga M, Harvey BL, Rossnagel BG, Scoles GJ, Chibbar RN (2003). Production of multiple shoots from thidiazuron-treated mature embryos and leaf-base/apical meristems of barley (*Hordeum vulgare*). Plant Cell Tissue Organ Cult.

[27] Ganie SA, Molla KA, Henry RJ (2019). Advances in understanding salt tolerance in rice. Theor Appl Genet.

[28] Genc Y, McDonald G, Tester M (2007). Reassessment of tissue Na + concentration as a criterion for salinity tolerance in bread wheat. Plant Cell Environ.

[29] Hadrami IE, Hadrami AE (2009). Breeding date palm. Breeding plantation tree crops: tropical species.

[30] Joshi P, Dhawan V (2007). Assessment of genetic fidelity of micropropagated *Swertia chirayita* plantlets by ISSR marker assay. Biol Plant.

[31] Kautz B, Hunsche M, Noga G (2014). Salinity-induced changes of multiparametric fluorescence indices of tomato leaves. Agriculture.

[32] Krishna H, Alizadeh M, Singh D, Singh U, Chauhan N, Eftekhari M, Sadh RK (2016). Somaclonal variations and their applications in horticultural crops improvement. 3 Biotech..

[33] Krivosheeva AB, Varlamova TV, Yurieva NO, Sobolkova GI, Kholodova VP, Belyaev DV (2014). Potato transformation with the HvNHX3 gene and the improvement of transformant salt tolerance. Russ. J. Plant Physiol..

[34] Kumar N, Modi AR, Singh AS, Gajera BB, Patel AR, Patel MP, Subhash N (2010). Assessment of genetic fidelity of micropropagated date palm (*Phoenix dactylifera* L.) plants by RAPD and ISSR markers assay. Physiol Mol Biol Plants.

[35] Lakshmanan V, Reddampalli VS, Neelwarne B (2007). Molecular analysis of genetic stability in long-term micropropagated shoots of banana using RAPD and ISSR markers. Electron J Biotechnol.

[36] Levy D, Veilleux RE (2007). Adaptation of potato to high temperatures and salinity-a review. Am J Potato Res.

[37] Liang W, Ma X, Wan P, Liu L (2019). Plant salt-tolerance mechanism: a review. Biochem Biophys Res Commun.

[38] López-Climent MF, Arbona V, Pérez-Clemente RM, Gómez-Cadenas A (2008). Relationship between salt tolerance and photosynthetic machinery performance in citrus. Environ Exp Bot.

[39] Lucia EHD, Whitehead D, Clearwater MJ (2003). The relative limitation of photosynthesis by mesophyll conductance in co-occurring species in a temperate rainforest dominated by the conifer *Dacrydium cupressinum*. Funct Plant Biol.

[40] Marashi SS, Hajilou J, Tabatabaei SJ, Nahandi FZ, Toorchi M (2017). Screening date palm cultivars for salinity tolerance using physiological indices. Pak J Bot.

[41] Martinez V, Nieves-Cordones M, Lopez-Delacalle M (2018). Tolerance to stress combination in tomato plants: new insights in the protective role of melatonin. Molecules.

[42] Meloni DA, Oliva MA, Martinez CA, Cambraia J (2003). Photosynthesis and activity of superoxide dismutase, peroxidase and glutathione reductase in cotton under salt stress. Environ Exp Bot.

[43] Moghaieb R, Abdel-Hadi A, Ahmed M (2011). Genetic stability among date palm plantlets regenerated from petiole explants. Afr J Biotechnol.

[44] Mohamed MA, Harris PJC, Henderson J (2000). In vitro selection and characterisation of a drought tolerant clone of *Tagetes minuta*. Plant Sci.

[45] Munns R, Tester M (2008). Mechanisms of salinity tolerance. Annu Rev Plant Biol.

[46] Murashige T, Skoog F (1962). A revised medium for rapid growth and bioassays with tobacco tissue cultures. Physiol Plant.

[47] Qiu N, Lu Q, Lu C (2003). Photosynthesis, photosystem II efficiency and the xanthophyll cycle in the salt-adapted halophyte *Atriplex centralasiatica*. New Phytol.

[48] Rasheed A, Xia X (2019). From markers to genome-based breeding in wheat. Theor Appl Genet.

[49] Rastogi J, Siddhant B, Sharma, B (2015). Somaclonal variation: A new dimension for sugarcane improvement. GERF Bull Biosci.

[50] Redondo-Gómez S, Wharmby C, Castillo JM, Mateos-Naranjo E, Luque CJ, de Cires A, Luque T, Davy AJ, Enrique Figueroa M (2006). Growth and photosynthetic responses to salinity in an extreme halophyte, *Sarcocornia fruticosa*. Physiol Plant.

[51] Roy SJ, Negrão S, Tester M (2014). Salt resistant crop plants. Curr Opin Biotechnol.

[52] SAS, 2011. SAS/STAT 9.3 User’s Guide. SAS Inst. Inc., Cary, NC.

[53] Sattar MN, Iqbal Z, Tahir MN, Shahid MS, Khurshid M, Al-Khateeb AA, Al-Khateeb SA (2017). CRISPR/Cas9: A practical approach in date palm genome editing. Front Plant Sci.

[54] Shabala S, Cuin TA (2008). Potassium transport and plant salt tolerance. Physiol Plant.

[55] Steel R, Torrie J (1980). Principals and procedures of statistics: a biometric approach.

[56] Sudhaa GS, Ramesh P, Sekhar AC, Krishna TS, Bramhachari PV, Riazunnisa K (2019). Genetic diversity analysis of selected Onion (*Allium cepa* L.) germplasm using specific RAPD and ISSR polymorphism markers. Biocat Agric Biotech.

[57] von Caemmerer S, Farquhar GD (1981). Some relationships between the biochemistry of photosynthesis and the gas exchange of leaves. Planta.

[58] Wahid A, Perveen M, Gelani S, Basra SMA (2007). Pretreatment of seed with H2O2 improves salt tolerance of wheat seedlings by alleviation of oxidative damage and expression of stress proteins. J Plant Physiol.

[59] Yaish MW, Kumar PP (2015). Salt tolerance research in date palm tree (*Phoenix dactylifera* L.), past, present, and future perspectives. Front. Plant Sci..

[60] Yaish MW, Patankar HV, Assaha DVM, Zheng Y, Al-Yahyai R, Sunkar R (2017). Genome-wide expression profiling in leaves and roots of date palm (*Phoenix dactylifera* L.) exposed to salinity. BMC Genomics.

[61] Zale JM, Borchardt-Wier H, Kidwell KK, Steber CM (2004). Callus induction and plant regeneration from mature embryos of a diverse set of wheat genotypes. Plant Cell Tissue Organ Cult.

